# Fertility preservation in pregnant cancer patients after first-trimester abortion: a new challenge with possible solutions

**DOI:** 10.1007/s10815-023-02950-5

**Published:** 2023-10-05

**Authors:** Loris Marin, Guido Ambrosini, Chiara Vio, Jordyn Conley, Luciana Bordin, Chiara Sabbadin, Alessandra Andrisani

**Affiliations:** 1 Department of Women’s and Children’s Health, University of Padua, Via Giustiniani 3, 35128 Padua, Italy; 2 Department of Molecular Medicine-Biological Chemistry, University of Padova, 35131 Padua, Italy; 3 Endocrinology Unit, Department of Medicine, University of Padova, 35128 Padua, Italy

**Keywords:** Fertility preservation, Oncofertility, Oocyte cryopreservation, Ovarian tissue cryopreservation, Post-abortion ovarian stimulation

## Abstract

Fertility preservation in pregnant women recently diagnosed with cancer can be a challenge. Raised levels of human chorionic gonadotropin (Beta-hCG) and progesterone in this population of patients may pose a problem for the prompt initiation of controlled ovarian stimulation (COS) due to a potential negative feedback of these hormones on folliculogenesis; however, it is not feasible to wait for negativization of serum beta-hCG levels before starting controlled ovarian stimulation. In literature, very few cases have been reported regarding the preservation of fertility in pregnant women recently diagnosed with cancer. We performed an extended revision of the literature to evaluate the current knowledge of the management of fertility preservation in women with cancer and we examined two cases closely. The first case study involved a cancer patient who underwent surgical abortion at 6.5 weeks of gestation followed by administration of mifepristone to detach any minimal residual trophoblast and consequently to decrease serum beta-hCG and progesterone levels before starting COS. In the second case study, the cancer patient underwent surgical abortion at 7.1 weeks of gestation and simultaneous unilateral oophorectomy for ovarian tissue cryopreservation due to a limited time for COS. By analyzing the results of these studies, it could be hypothesized that mifepristone administration may favor the decrease of serum beta-hCG and progesterone levels in order to permit rapid initiation of COS. In cases where COS is not feasible, ovarian tissue cryopreservation should be considered as an alternative fertility preservation technique.

## Background

Hematological cancers and breast cancers are among the most common oncological diseases diagnosed in adolescents and young adults and their associated treatments can negatively impact fertility. Due to the diagnosis of such tumors at younger ages and the trend of delaying motherhood until older ages [1], a rising number of women are diagnosed with cancer during their childbearing ages and an additional group are diagnosed with cancer during pregnancy. These women must be counseled regarding the risks of continuing the pregnancy, as many cancer treatments are contraindicated during pregnancy, as well as regarding the risk of infertility caused by cancer treatments and available options for fertility preservation [2–5]. COS for oocyte/embryo cryopreservation is the first-choice of treatment for fertility preservation, and ovarian tissue cryopreservation should be considered when COS is not feasible [2–5]. In the literature some studies report fertility preservation treatment outcomes in pregnant women recently diagnosed with cancer who have decided to terminate the pregnancy. Raised beta- hCG levels should be considered as they may interfere with controlled ovarian stimulation (COS) outcome; however, it is not always feasible to wait until hCG levels are negative before initiating treatment, as it is of primary importance to retrieve mature oocytes without delaying the start of cancer treatments [6, 7]. The aim of our study is to review available publications regarding this topic and evaluate a possible solution for this challenge. Administration of Mifepristone to rapidly decrease serum hCG and progesterone levels may improve oocyte yield in controlled ovarian stimulation after pregnancy termination.

## Material and methods

A review of studies was performed evaluating the management of fertility preservation in pregnant women recently diagnosed with cancer. A comprehensive review of the literature was performed using PubMed, Scopus, Embase, ScienceDirect and Google Scholar up to March 2023. Key search terms included the following: “fertility preservation” AND “hCG” OR “abortion” OR “pregnancy termination” and “ovarian tissue cryopreservation” AND “hCG” OR “abortion” OR “pregnancy termination.” Inclusion criteria consisted of studies reported in English regarding women undergoing fertility preservation for oncological reasons after first-trimester abortion. To avoid confounding mechanisms due to different hormonal levels, reports about fertility preservation beyond the first trimester or in cancer patients who recently gave birth, were excluded. In addition, we reported two unpublished cases of fertility preservation in oncological patients who presented in early pregnancy.

## Results

The electronic searches provided a total of 877 citations, after the removal of 60 duplicate records, 817 citations remained. Of these, 811 records were excluded after title/abstract screening (not relevant to the review) and 6 manuscripts were included in the review. These manuscripts report six cases of COS for fertility preservation in cancer patients after first-trimester abortion with varying outcomes [6–11] (Table 1). The details have been reported elsewhere [6–11]. Briefly, in three cases the fertility preservation treatment outcome was shown to be negative or suboptimal ranging from no oocytes retrieved [7], retrieval of 3 three degenerated oocytes [6], or retrieval of mostly immature oocytes or low-quality oocytes [10]. The serum hCG levels at the start of treatment in these cases were 5000 UI/L and 116,420 IU/L, respectively, and 17,867 IU/L detected on day 8 of stimulation. In the case of the patient where only degenerated oocytes were obtained, a second COS was performed with an hCG level of 69.3 IU/L at the start of the treatment which ultimately led to retrieval of 8 mature oocytes [6]. Adequate outcomes were achieved in three cases in which 28 [9], 29 [11] and 18 oocytes [8] were retrieved and the hCG levels were 5,600 UI 3 days before the start of controlled ovarian stimulation, 119.8 and 222 at the start of the treatment, respectively. Only one case reports a patient who underwent both COS and ovarian tissue harvesting [9].

**Table 1:**
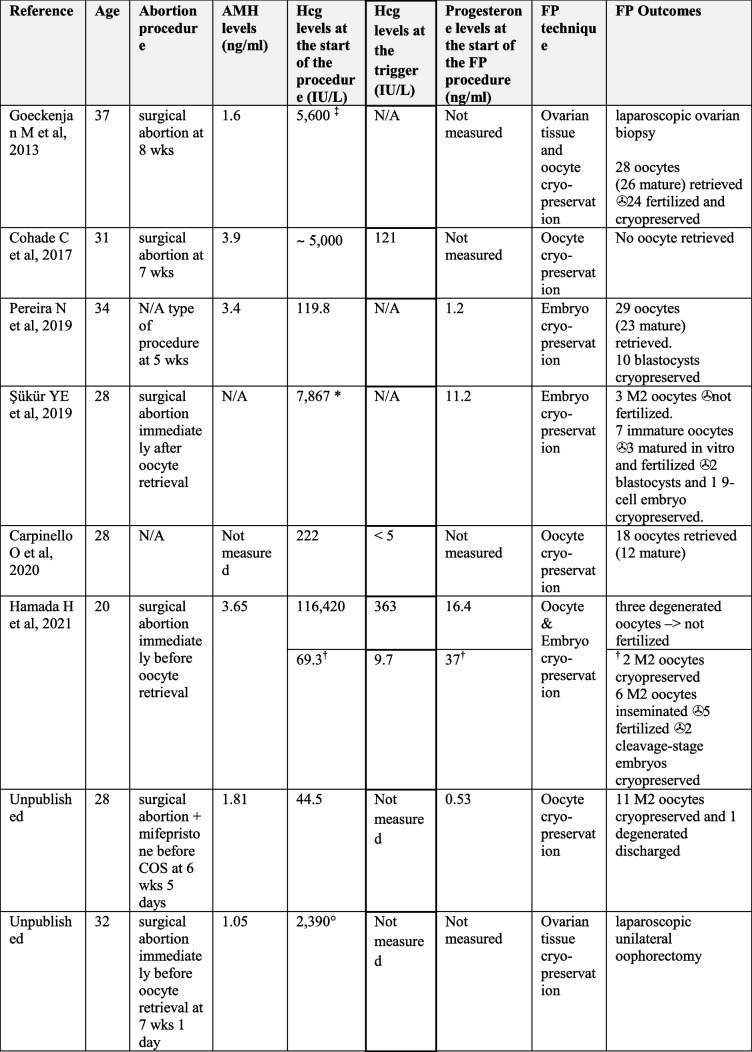
Data about features of the cases reported in the literature including the Hcg and progesterone levels at the start of the fertility preservation procedure and fertility preservation outcomes

^‡^ 5 days after abortion, 2 days before laparoscopic ovarian biopsy and 3 days before the start of controlled ovarian stimulation

* detected on day 8 of stimulation

^†^ second controlled ovarian stimulation

° 14 days before FP procedure

We chose to include two unpublished cases in our report. To our knowledge, this is the first reported case regarding fertility preservation after a combined medical and surgical abortion, as well as the first reported case of fertility preservation with cryopreservation of ovarian tissue alone in a pregnant woman with cancer (Table 1). The first patient (Patient A) underwent COS and oocyte cryopreservation after dilatation and curettage (D&C) for abortion. In order to lower serum human chorionic gonadotropin (hCG) levels, Mifepristone (RU-486) 600 mg was administered. Oocyte retrieval was then successfully performed and chemotherapy treatment was started. The second patient (Patient B) underwent a combined surgery of D&C and laparoscopy for unilateral oophorectomy for ovarian tissue cryopreservation, due to a limited time for COS. The patients signed informed consent for the procedures they underwent, as well as to allow data collection for research purposes and the publication of these case reports. Considering the anonymized data collection and description of the cases, a formal Institutional Review Board approval was exempted.

### Patient A

**Fig. 1:**
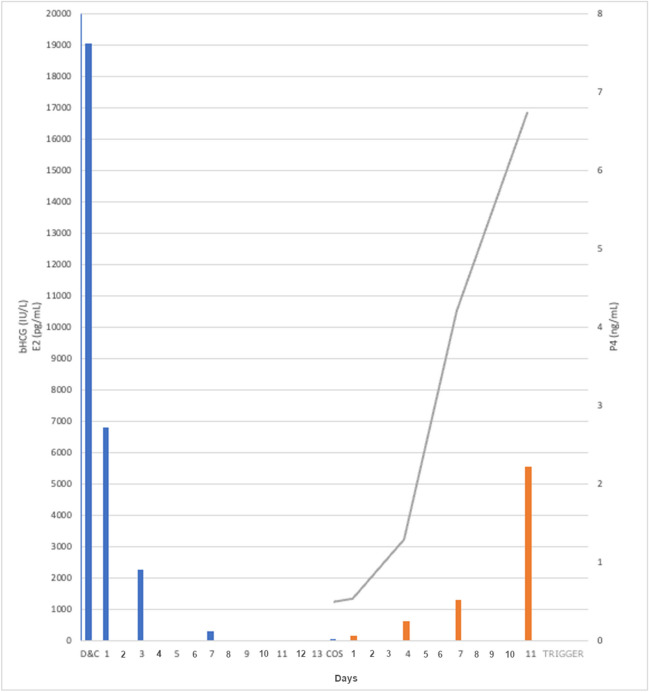
Change of hormone levels after abortion and during ovarian stimulation. Blue bars: beta-Hcg levels, orange bars: estradiol levels, gray line: progesterone levels. Horizontal bar: days from D&C and days from COS (controlled ovarian stimulation) starting

A 28-year-old woman presented to our hospital with night sweats and itching for a period of two months. No other symptoms were reported. Prior to this occasion her personal medical history had been unremarkable. Fine-needle aspiration of a two centimeter-large cervical lymph node was performed and ultimately a diagnosis of Classic Hodgkin Lymphoma, stage IIA was determined. The diagnostic pathway was then completed with an excisional lymph node biopsy, a total-body computerized tomography and a positron emission tomography. Hematologists indicated initiation of a chemotherapy protocol with doxorubicin, bleomycin, vinblastine, and dacarbazine (ABVD) within a month from the first fertility preservation consultation. While performing a complete lab assessment before beginning COS, high levels of serum beta-hCG were detected (5959 U/L). A transvaginal ultrasound was then performed, which confirmed a pregnancy dated at 6.1-weeks. After a multidisciplinary discussion with hematologists and gynecologists, the patient was informed about the risks of continuing the pregnancy and the existing fertility preservation techniques were evaluated. The woman opted for pregnancy termination, and a surgical abortion procedure was successfully performed at 6 weeks 5 days of gestation. On the day of the D&C procedure, beta-hCG levels were 19064 U/L. The patient received Mifepristone 600 mg (RU486) as an off-label treatment after providing informed consent. The day after RU486 administration, serum beta-hCG levels were 6802 U/L, while 2 days after serum levels were 2258 U/L and 6 days after drug administration, beta-hCG levels further dropped to 308.9 U/L (Fig. 1) and progesterone levels were 0.79 ng/ml. Two weeks after termination of the pregnancy, the patient began COS. At the beginning of COS, hCG-levels were 44.5 U/L and progesterone levels were 0.53 ng/ml. AMH levels were 1.81 ng/ml. The patient received 3,300 IU recombinant follicle stimulating hormone (Ovaleap, Theramex Italy) according to the Gonadotropin-Releasing Hormone (GnRH)-antagonist short protocol. After 11 days of COS, the patient developed 14 follicles ≥ 17 mm and subsequently ovulation was induced following injection of 0.3 mg triptorelin acetate as a GnRH agonist. Oocyte retrieval was performed after 35.5 h with retrieval of 12 oocytes (11 mature and one degenerated) followed by cryopreservation (Table 1). Few days after oocyte retrieval, the patient began chemotherapy and underwent two cycles of ABVD. Two months after oocyte retrieval, positron emission tomography showed a complete metabolic response to treatment. At the 6-month follow-up the patient reported subjective well-being and blood exams and instrumental tests were normal.

### Patient B

A 32-year-old woman was referred to our regional fertility preservation center following diagnosis of an invasive ductal carcinoma, hormone-receptor positive. The patient underwent a left quadrantectomy and axillary lymph-node dissection, which resulted positive upon histological examination, (stage IIA), followed by adjuvant chemotherapy with Epirubicin, Cyclophosphamide and Paclitaxel. Her gynecological history included two vaginal deliveries in 2007 and 2021. During fertility preservation workup, the patient stated that her last menstrual period had occurred eleven days before the consultation. Five antral follicles were detected during ultrasound examination. The woman underwent blood sampling to measure serum beta-hCG levels and AMH levels. Beta-hCG levels were 2390 U/L and AMH levels were 1.05 ng/ml. The patient was informed in a multidisciplinary discussion with oncologists and gynecologists about the risks of continuing the pregnancy. Two weeks after diagnosis of the pregnancy, the woman opted for pregnancy termination. The possible abortion procedures and fertility preservation techniques were evaluated. The woman successfully underwent a surgical abortion procedure at 7 weeks and 1 day and laparoscopic right ovariectomy for ovarian tissue cryopreservation contextually. Ovarian tissue was cryopreserved with the slow freezing method (Table 1). A sample was histologically analyzed to rule out the presence of cancer cells and for follicle count: primordial follicle density was 7 follicles/mm^3^ and primary follicle density was 2 follicles/mm^3^. Two days after D&C and unilateral oophorectomy the patient started chemo, followed by quadrantectomy after eight weeks.

## Discussion

In order to highlight the risk of infertility after undergoing gonadotoxic therapies often used in treatment of oncological diseases, international societies of oncology and reproductive medicine recommend offering counseling to cancer patients of childbearing age with favorable prognosis regarding available methods of fertility preservation [5, 12–15]. The first line of treatment for fertility preservation is oocyte/embryo cryopreservation when feasible [5]. Although ovarian tissue transplantation is no longer considered experimental [5] the procedure requires at least two surgeries [2, 16]. It is relatively uncommon for women to be diagnosed with cancer during pregnancy; thus, available data regarding fertility preservation in pregnant women recently diagnosed with cancer are limited and further studies are needed. The early stages of pregnancy are characterized by an increase of hormones including beta-hCG and progesterone. These hormones may have a negative impact on ovarian stimulation and oocyte retrieval. It is not clear whether high levels of hCG and/or progesterone can impair folliculogenesis, and thus oocyte retrieval. In literature, only a few case reports have been published regarding COS for fertility preservation in patients with elevated hCG levels after first-trimester abortion and overall they present conflicting results [6–11]. After a literature review, we noticed that when hCG levels are high, folliculogenesis seems to be impaired as described by Cohade et al. [7]. Additionally, in some cases oocytes could not be retrieved, as described by Sukur et al. [10], in which only 3 mature oocytes and 7 immature oocytes were retrieved. Fertilization of the mature oocytes obtained was not achieved. In a case described by Hamada H et al. [6] 3 degenerated oocytes were retrieved after the first COS. The woman underwent a subsequent COS resulting in retrieval of 8 mature oocytes. Despite the improved result, the outcome of IVF treatment is still not optimal considering the age of the patient and her ovarian reserve [6].

Only the case reported by Goeckenjan M et al. [9] evidenced an optimal oocyte yield despite high hCG levels. Goeckenjan et al. [9] also hypothesized that the presence of serum hCG during COS might increase the number of oocytes yielded through a LH-like action; However, in this particular case, the day before the start of COS the patient underwent laparoscopic ovarian biopsy for ovarian tissue cryopreservation. It cannot be ruled out that the corpus luteum may have been removed during surgery with consequent drop of progesterone levels that can promote trophoblast detachment and consequently a rapid decrease of hCG levels; in fact, levels were 300 IU/l on day 5 of COS and 150 IU/l on day 7 of COS.

Hamada et al. [6] and Cohade et al. [7] hypothesized different reasons for the failure of folliculogenesis, such as the premature luteinization of the follicle caused by beta-hCG bound to luteinizing hormone (LH) receptors on the granulosa cells, or the ineffective LH surge caused by the saturation of LH receptors by beta-hCG, and the high level of progesterone that prevents the surge of LH after injection of GnRH agonists.

Controlled ovarian stimulation outcomes were optimal when hCG levels were low; in fact, in the cases reported by Carpinello et al. [8] and Pereira et al. [11] the number of retrieved oocytes was optimal with hCG levels before the start of treatment measuring 222 IU/l and 119.8, respectively. In the case of patient A, hCG levels were low at the beginning of COS and the number of mature retrieved oocytes resulted adequate considering the patient's ovarian reserve. For this patient, the hematologist planned to start chemotherapy within a month following our first consultation and we scheduled the start of COS about 2 weeks before the beginning of the oncological treatment. A surgical approach through D&C was decided for the patient rather than medical treatment due to time limitations. D&C is a relatively quick solution in this case; however, it is important to proceed with caution in order to avoid the development of synechia that can compromise future fertility [17]. After a D&C, small parts of trophoblast may be retained and continue to secrete beta-hCG; however, they are often discharged with the next menstruation. For this reason, the patient was administered mifepristone 600 mg immediately after D&C. Mifepristone is a synthetic steroid that acts as a competitive progesterone receptor antagonist. This drug is usually used for medical abortion to block progesterone receptors in the endometrium and thus to promote detachment of the trophoblast and consequently a decrease in hCG secretion which impairs the corpus luteum and its progesterone secretion [17]. The administration of mifepristone immediately after D&C can contribute to detachment of the remaining trophoblast and to further decrease serum beta-hCG and progesterone levels. It has been highlighted that there is a rapid decline in serum hCG levels after medical abortion with the use of mifepristone [18, 19], even if the precise action of mechanism of hCG decline has not yet been elucidated [20]*.* Despite the fact that timing of ovulation after abortion is wide, with a range of 6–103 days, serum hCG levels become undetectable in approximately 38 days after D&C [21]. In this case, hCG serum levels rapidly dropped with a decrease of 88.2% in 3 days (from 19,064 to 2258 U/l) and a decrease of 98.4% in 1 week (from 19064 to 308.9 U/l). Two weeks after D&C, when the patient started COS, her hCG serum levels were very low (44.5 U/l), although not negative, and progesterone levels were 0.53 ng/ml.

A multidisciplinary approach is essential for the management of women undergoing fertility preservation, in particular for women who are pregnant at the time of the diagnosis of cancer. It is speculated that early luteinization may be prevented if hCG levels are less than 20 mIU/mL at the time of follicle development [6, 7]. Adequate oocyte retrieval has been performed with hCG levels up to 222 IU/l at the start of the treatment [8]. Gynecologists managing these patients should schedule the start of COS to perform it successfully within a short time frame in accordance with oncologists/hematologists in order not to delay the start of chemotherapy [5, 22]. Other studies are needed to confirm our hypothesis that mifepristone may accelerate the decrease of hCG and progesterone levels and therefore COS may be started sooner.

In our second case, patient B was counseled about the risks of chemotherapy administration in the first trimester and the necessity of starting chemotherapy within 8 weeks following quadrantectomy [4]. Since the patient was counseled many times before she could make her decision about abortion, we did not have enough time to undergo D&C first and COS secondly; therefore, we performed a laparoscopic unilateral oophorectomy for ovarian tissue cryopreservation. Moreover, a single ovarian stimulation might not have been sufficient for the retrieval of an optimal number of oocytes due to her diminished ovarian reserve. We cannot rule out that the disruption of the Hippo-signaling pathway that occurs during cortical ovarian tissue preparation for cryopreservation may reactivate dormant follicles, as described in the Drug-Free in vitro activation [23].

It is evident that there are many factors in the decision-making process for young women scheduled for gonadotoxic therapy who are considering fertility preservation. When the diagnosis of an oncological disease is received during early pregnancy, stress levels are significantly higher as the patient must make important decisions regarding abortion. Unlike infertile couples who exhibit the ability to deal with the unexpected (dyadic coping) [24], during fertility preservation treatments the choice is left up to the patient individually. This can be difficult even for patients who have an adequate social support system. For these reasons, in the event of a scheduled oophorectomy for ovarian cryopreservation, it is suggested to perform D&C and laparoscopy simultaneously in order to reduce the number of times the patient must undergo anesthesia and also to reduce patient stress levels.

In the case of the removal of ovarian tissue for cryopreservation, it is ideal to avoid the couple having to relocate to a center specialized in fertility preservation. If the reference center for the cryopreservation of ovarian tissue does not offer the possibility of abortion, or vice versa, in order to reduce patient stress and avoid multiple surgeries, it is possible to perform retrieval of ovarian tissue (biopsy or ovariectomy) in any center and send the specimens to the referral fertility center within several hours.

In conclusion, the need to start fertility preservation therapy immediately after an interruption of pregnancy is challenging. From available data in the literature and the two cases described, hCG levels may affect stimulation outcomes. In fact, just one case report in the literature hypothesized that beta-hCG can improve the outcome of COS through a *LH-like* action [9], and other reports showed a detrimental effect of beta-hCG on COS outcomes [6, 7, 10]. Until further studies demonstrate the exact mechanism of action of beta-hCG on folliculogenesis, strategies to rapidly decrease beta-hCG levels are needed. When there is insufficient time to wait for beta-hCG levels to drop, ovarian tissue cryopreservation should be considered.

## Data Availability

All available data has been reported in the manuscript.
